# Hemothorax as a complication of transbronchial lung cryobiopsy

**DOI:** 10.1002/rcr2.1034

**Published:** 2022-09-05

**Authors:** Haruhiko Michimata, Toshiyuki Sumi, Yoshiko Keira, Daiki Nagayama, Yuta Koshino, Hiroki Watanabe, Yuichi Yamada, Yusuke Tanaka, Hirofumi Chiba

**Affiliations:** ^1^ Department of Pulmonary Medicine Hakodate Goryoukaku Hospital Hakodate Japan; ^2^ Department of Respiratory Medicine and Allergology Sapporo Medical University School of Medicine Sapporo Japan; ^3^ Department of Surgical Pathology Hakodate Goryoukaku Hospital Hakodate Japan

**Keywords:** computed tomography, hemothorax, intravascular lymphoma, transbronchial lung cryobiopsy

## Abstract

There are more complications in transbronchial lung cryobiopsy than in a conventional transbronchial lung biopsy. Respiratory endoscopists should be aware of the potential complications, including rare complications such as hemothorax.

## Graphical abstract

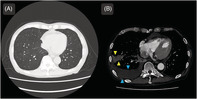

## CLINICAL IMAGE

**FIGURE 1:**
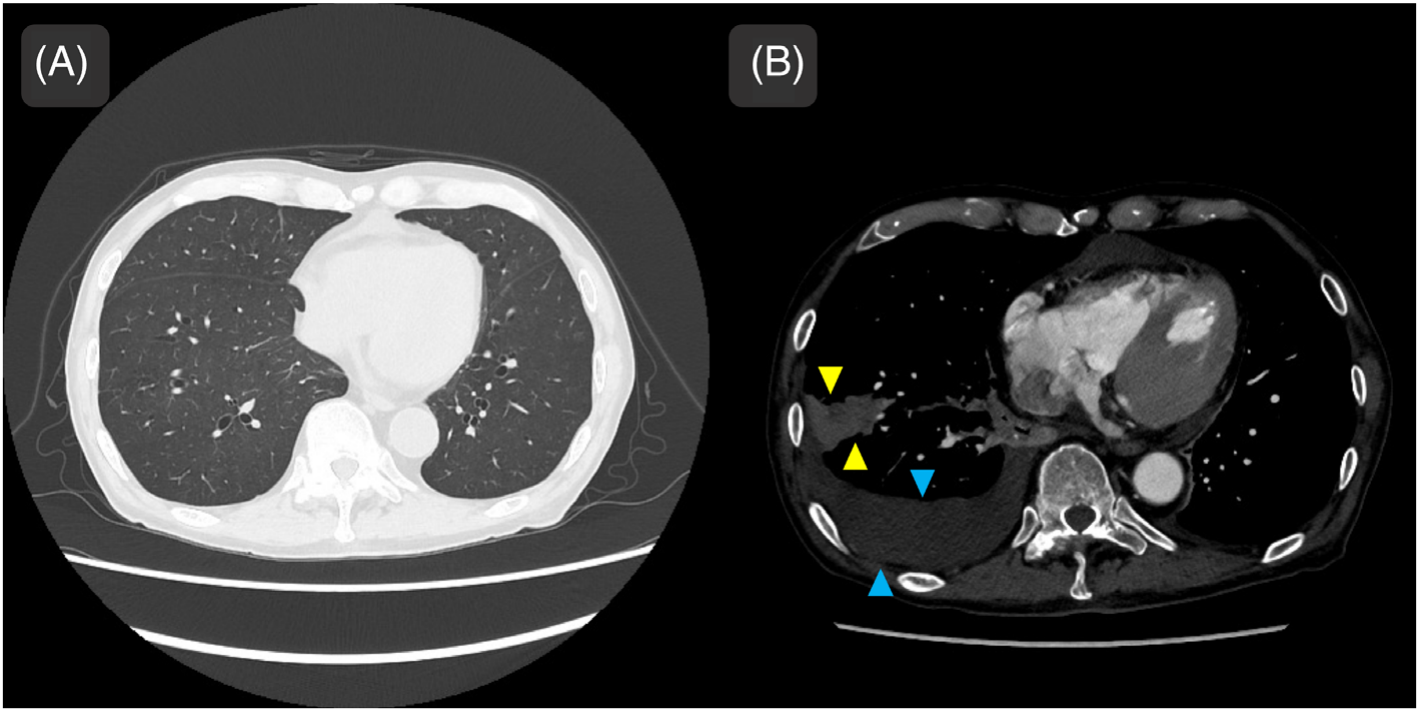
Imaging findings. (A) Chest CT shows bilateral diffuse ground‐glass opacities. (B) Contrast‐enhanced chest CT after transbronchial lung cryobiopsy shows a wedge‐shaped infiltrating shadow (yellow arrow head indicated) in the right lower lobe and a right hemothorax (blue arrow head indicated). There is no evidence of active haemorrhage. CT, computed tomography

**FIGURE 2:**
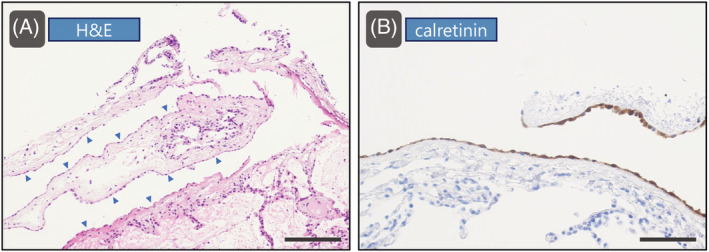
Pathological findings. (A) H&E stained section of the lung sample shows the visceral pleura (indicated by blue arrowheads). The scale bars represent 100 μm. (B) Mesothelial cells in the visceral pleura are positive for calretinin. The scale bars represent 50 μm. H&E, haematoxylin and eosin

A man in his 80s visited our hospital with fever and dyspnea. Chest computed tomography (CT) revealed diffuse ground‐glass opacities in both lungs (Figure 1A). Intravascular lymphoma was suspected based on laboratory findings. Fluoroscopic transbronchial lung cryobiopsy (TBLC) of the right lower lobe was performed twice. Moderate intraoperative bleeding occurred, which was endoscopically controlled. Although the patient was asymptomatic, chest radiographs obtained 1 h postoperatively showed a right pleural effusion. Contrast‐enhanced CT revealed a hemothorax (Figure 1B). Since there was no pneumothorax or active bleeding, the patient was treated conservatively with transfusion; no persistent bleeding occurred. Histopathological examination revealed mesothelial cells positive for calretinin, indicating visceral pleura was sampled (Figure 2).

Complication rates are higher in TBLC than in conventional biopsy in patients with interstitial lung diseases, with pneumothorax and moderate/severe bleeding rates of 12% and 39%, respectively.^1^ However, TBLC‐induced hemothorax is rare.^2^ In this case, sampling of the visceral pleura resulted in pleural damage; however, no pneumothorax was observed. The airway proximal to the biopsy site might have been obstructed during endoscopic haemostasis, resulting in a hemothorax from bleeding into the pleural cavity or distal to the parenchyma. Consequently, a blood patch may have formed preventing a pneumothorax.

## AUTHOR CONTRIBUTION

Toshiyuki Sumi is the guarantor of the clinical content of this submission

## CONFLICT OF INTEREST

None declared

## ETHICS STATEMENT

The authors declare that appropriate written informed consent was obtained from the patient for the publication of this report and any accompanying images

## Data Availability

The data that support the findings of this study are available from the corresponding author upon reasonable request.
